# A comprehensive annotated image dataset for deep learning analysis of eggplant leaf diseases

**DOI:** 10.1016/j.dib.2025.112140

**Published:** 2025-10-08

**Authors:** Md. Asraful Sharker Nirob, Prayma Bishshash, Mariyam Bin Ayan, Tania Khatun, Shayla Sharmin, Md Zahid Hasan, Mohammad Shorif Uddin

**Affiliations:** a Health Informatics Research Lab, Department of CSE, Daffodil International University, Daffodil Smart City, Birulia, Savar, Dhaka 1216, Bangladesh; b Department of Computer Science and Engineering, Jahangirnagar University, Savar, Dhaka 1342, Bangladesh

**Keywords:** Eggplant leaf disease, Agricultural disease detection, Plant pathology, Image dataset, Machine learning in agriculture deep learning

## Abstract

The Eggplant Leaf Disease Dataset was meticulously developed to address challenges in accurately identifying diseases that threaten eggplant crops, a vital agricultural resource worldwide. This dataset includes 3116 high-resolution images captured between March and May 2024 from two major agricultural regions in Bangladesh, representing real-world conditions. It comprises 10 distinct disease classes—Aphids, Cercospora Leaf Spot, Defect Eggplant, Flea Beetles, Fresh Eggplant, Fresh Eggplant Leaf, Leaf Wilt, Phytophthora Blight, Powdery Mildew, and Tobacco Mosaic Virus—making it the most comprehensive dataset for eggplant diseases to date. To enhance its utility, rigorous data augmentation techniques, including flipping, rotating, shearing, shifting, noise addition, and brightness adjustment, were applied. This expanded the dataset to 10,000 images, ensuring its robustness for machine learning applications. Expert annotations further enhance its quality, providing critical insights for precise disease classification.

Our Proposed CBAM–EfficientNetB0 model had an amazing classification accuracy of 98.70 %, which was much better than the baseline architectures. ResNet50 only got 32.60 %, VGG16 got 73.00 %, and VGG19 got 68.00 %. The proposed model's better performance shows that combining channel and spatial attention through CBAM with EfficientNetB0′s feature extraction abilities works well. This architecture does a good job of picking out the distinguishing features in eggplant leaf images, which makes it possible to accurately identify diseases. The dataset and model work together to make AI-powered early disease detection, automated monitoring, and decision support in precision agriculture possible. These tools help farmers use sustainable farming methods by making timely interventions, reducing the need for manual inspection, and increasing crop productivity and food security.

Specifications Table

**Table:** 

Subject	Computer Science
Specific subject area	Smart Farming, Classification of Eggplant leaf, Diseases Image Classification, Plant Pathology, Agricultural Informatics, Plant Disease Detection, Image Recognition, Computer Vision, and Deep Learning in Agricultural Applications
Type of data	Image.
Data collection	The dataset collected from March to May 2024 in Dattapara, Ashulia, Dhaka, Bangladesh, Gazaria, Munshigonj, Dhaka, Bangladesh, and Horinadundu, Jhenaidah Sadar, Bangladesh, comprises 3116 high-resolution (800 × 800 pixels) images of eggplant leaves, each expertly annotated to classify 10 diseases. Despite challenges from adverse weather, stringent quality control measures ensured dataset integrity. This resource is crucial for advancing plant pathology research and developing AI-driven disease detection tools. Special thanks to Mohammad Enayet-e-Rabbi, Deputy Director of Quality Control at the Seed Certification Agency, Ministry of Agriculture, Bangladesh, for his invaluable feedback and cooperation*.*
Data source location	Town/City/Region: Dhaka, Musnshigonj and Jhenaidah Sadar. Country: Bangladesh
Data accessibility	Repository name: Mendeley Data Data identification number: 10.17632/5drkk544k8.1 Direct URL to data: https://data.mendeley.com/datasets/5drkk544k8/1
Related research article	*none.*

## 1 Value of the Data

• This dataset stands out from existing collections by offering the largest number of eggplant leaf disease classes to date, covering a wide range of common fungal, bacterial, viral, and pest-related conditions. Further, a set of sophisticated image processing was performed to enhance its utility: flipping, rotation, Gaussian noise, shifting height, shifting brightness, zooming, and shearing. This augmented dataset would ensure comprehensiveness in the variations of the disease. The more robust and diverse dataset will serve as a better starting point for training deep learning models toward high accuracy and scalability, thus providing a better source for agricultural AI.
• Efficient and accurate disease detection is essential for sustainable agriculture. This dataset provides a foundation for developing AI-driven solutions that ensure early and accurate detection of crop diseases. These advancements empower farmers to act promptly, minimize losses, and improve overall crop yields. By providing a powerful resource for developing these tools, the dataset fosters technological advances that promote sustainable farming practices and improve agricultural productivity.
• Integrated with IoT devices and drone systems, such as UAVs fitted with high-resolution cameras and real-time image analysis capabilities, the dataset thus enables automated image capture and disease detection over large areas of agricultural fields. This integration thus allows early intervention, reduces manual labor, and optimizes resource usage. For instance, drones can efficiently keep track of crop health, find out the affected areas, and deliver targeted treatments, hence greatly improving operational efficiency in precision agriculture. The dataset contains high-resolution images of eggplant leaves, carefully annotated to identify various diseases with accuracy. Each image is labeled with detailed information on disease type, affected area and severity. These high-quality annotations are crucial for training and validating deep learning models, ensuring accurate disease detection and classification. Detailed labeling reduces noise in training data, increases model performance and reliability in practical applications. By providing precise annotations, the dataset enables the development of powerful machine learning algorithms that can distinguish between multiple diseases, even those with subtle differences in visual symptoms. This specificity is essential for developing models that can be deployed in real-world agricultural settings where accurate disease detection is critical for timely and effective intervention.
• This dataset is a significant resource for building and testing advanced machine learning models, especially deep learning architectures such as Convolutional Neural Networks (CNNs). Researchers can use this dataset to explore and refine algorithms for automated disease detection, pushing the boundaries of current methods. Its comprehensive nature allows for rigorous scientific studies, promotion of innovation and development of specific diagnostic tools that can be adapted to different agricultural contexts beyond eggplant leaf diseases. The availability of such datasets supports comparative studies, algorithm benchmarking, and the development of new techniques in computer vision and machine learning. By providing a strong foundation, the dataset accelerates research and development efforts aimed at improving crop health monitoring and management.

## 2 Background

The development of this dataset was driven by the need to tackle significant challenges in accurately identifying diseases affecting eggplant leaves, which are common issues in agriculture. This initiative is part of broader efforts in precision agriculture, which aims to improve crop management through technological advancements. The existing datasets for agricultural AI, in particular eggplant leaf diseases, have key limitations: low sample diversity in disease class and size, and poor quality in annotations. These limitations may directly affect the performance and generalizability of the models when applied to practical scenarios. Most of the existing datasets capture images against uniform white backgrounds, which by no means represent the natural field conditions in which diseases occur. Lacking this diversity in environmental context, the model generalization is more difficult for the AI models under real-world agricultural scenarios. Our dataset is unique compared to previous works. For example, M. Hasan et al. [1] presented 392 images augmented to 3136 images, but its limited diversity in disease classes and reliance on basic augmentation techniques hamper its real-world applications. Shakib Howlader et al. [2] proposed a dataset consisting of 4089 images of six disease classes. However, the white background on which images are captured is uniform and does not reflect natural conditions; hence, its applicability in the real world is limited. Our dataset consists of a larger number of high-resolution images and classes collected from two different locations and compared to the studies mentioned, which were collected from two different locations and. This increases the variability of the dataset, making it more representative of real-world agricultural environments and hence better generalizing for AI-driven research.

Moreover, this data article will support its related research publication with raw data that can be shared with researchers and practitioners to enhance transparency and reproducibility of research for further studies to find the best possible agricultural practices. In this way, this study contributes in the development of robust AI tools that can significantly improve disease management and increase productivity in eggplant farming.

## 3 Data Description

The dataset was collected over the period from March to May 2024 in three diverse locations: Dattapara, Ashulia, Dhaka, Bangladesh, and Horinadundu, Jhenaidah Sadar, Bangladesh. These regions were selected for their significance in eggplant cultivation, providing a comprehensive representation of common agricultural environments. The dataset consists of 3116 high-resolution images of eggplant leaves, each with a resolution of 800 × 800 pixels, captured using digital cameras and smartphones with high-quality lenses. Each image in the dataset has been meticulously annotated by domain experts to ensure precise identification and classification of various eggplant leaf diseases.

During the data collection phase, numerous challenges arose, primarily due to adverse weather conditions. The unusually cold weather posed difficulties in capturing clear images, resulting in noisy backgrounds and uneven lighting conditions. However, stringent quality control measures were enforced to safeguard the integrity and reliability of the dataset. Despite these challenges, images were diligently captured under diverse lighting conditions to emulate real-world scenarios. Additionally, random sampling and cross-validation techniques were employed to validate the accuracy of annotations, ensuring consistency and accuracy across the dataset. The dataset is meticulously formatted to facilitate seamless integration with machine learning frameworks, providing images in standard formats like JPEG. 1000 images per class were augmented The methods included were designed to account for variability in leaf orientation, lighting, and progressive (disease) severity or cover, providing a representative dynamic range of conditions. To ensure that augmented images contained disease-specific features without introducing artifacts, annotations were verified through cross-validation with rigorous manual review by domain experts.

**Fig. 1:**
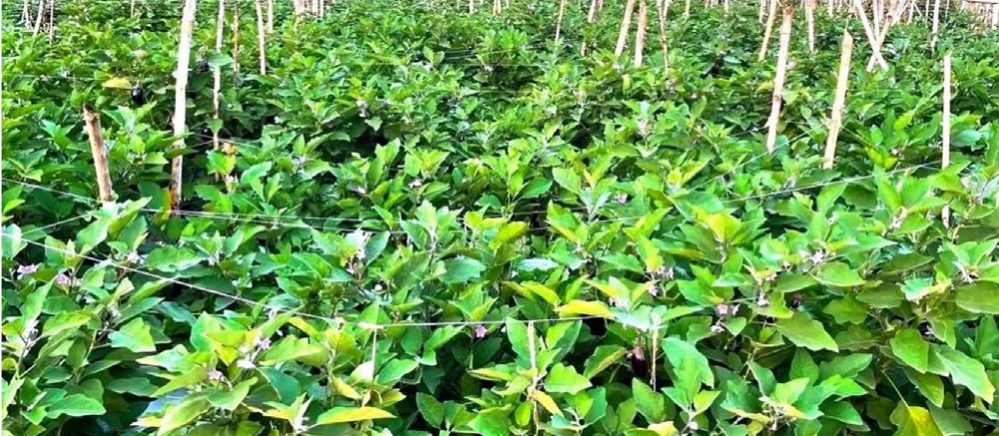
The real eggplant field where images in the dataset were collected.

To illustrate the data collection process, Fig. 1 showcases the field conditions under which the images were captured. The dataset was collected under various lighting and environmental conditions to ensure robustness and real-world applicability. On purpose, the data collection included cloudy skies and other unpredictable lighting conditions. These variations help emulate real-world challenges that affect image quality and disease visibility, enhancing the robustness of the dataset. By incorporating a wide range of environmental conditions, the dataset better reflects the variations that might be encountered in practical applications; hence, machine learning models trained on this data can generalize well to real-world scenarios. This holistic approach lets the dataset capture the symptoms of diseases under different lighting and environmental influences more accurately, thus making it more reliable for training AI-powered disease detection tools.

This comprehensive dataset is designed to propel advancements in plant pathology research and support the development of AI-driven disease detection tools. By encompassing a wide range of diseases including Aphids, Cercospora Leaf Spot, Defect Eggplant, Flea Beetles, Fresh Eggplant, Fresh Eggplant Leaf, Leaf Wilt, Phytophthora Blight, Powdery Mildew, and Tobacco Mosaic Virus, it caters to various research needs. Each disease class is comprehensively detailed in Table 1, providing crucial insights into their characteristics. Annotations accompanying the images furnish intricate information regarding the type of disease, affected areas, and the severity of infection, rendering the dataset indispensable for training and validating deep learning models dedicated to disease detection and classification.

**Table 1:** Brief description of the eggplant dataset.

Class Name	Description	Visualization
Cercospora Leaf Spot	Cercospora Leaf Spot in eggplants starts as small, round, yellow, slightly sunken spots [3] on the upper side of the older, lower leaves. These spots enlarge and become more irregular, surrounded by a yellow halo. As the disease progresses, the spots merge, turn brown, and appear on both leaf surfaces. This infection [4] can cause the older leaves to curl and drop off prematurely.	
Flea Beetles	Flea beetles infest eggplant leaves by feeding on the undersides of foliage and stems, creating tiny holes and pits resembling shotgun damage. This feeding causes the leaves to yellow and can stunt plant growth [5]. Young eggplants are particularly vulnerable, while more mature plants usually survive.	
Phytophthora Blight	Phytophthora Blight appears as water-soaked, light-brown spots on eggplant leaves, often causing premature leaf drop and defoliation. This disease, typically caused by Phytophthora capsici or P. nicotianae [8], may also result in dark streaks on the stems and branches. If untreated, it can cause rapid wilting and ultimately death plant [9].	
Powdery Mildew	Powdery Mildew appears as white, powdery growth on the underside of eggplant leaves [10]. Infected leaves may curl upward, and lesions may merge, causing yellowing and premature leaf drop. The disease starts on older leaves and spreads to younger ones if left untreated.	
Tobacco Mosaic Virus	The Tobacco Mosaic Virus (TMV) affects eggplant leaves by causing a distinctive mottling of light and dark green, which gives the leaves a variegated appearance. In addition to mottling, infected leaves can become thick, wrinkled, and distorted [11]. TMV is a widespread problem in plants of the Solanaceae family, which includes eggplants, tomatoes, and peppers. Managing TMV involves preventive measures such as using virus-free seeds, practicing good hygiene to prevent transmission, and removing and destroying infected plants to reduce spread within the crop [12].	
Aphids	Aphids are small, soft-bodied insects usually green or brown, identified by their long antennae and distinctive cornicles at their rear, which secrete a waxy substance. When they infest eggplant leaves, aphids typically gather at the growth points and the undersides of new leaves. Their presence causes several harmful symptoms [13], including leaf spotting, yellowing (chlorosis), curling, and distortion. In severe cases, they can cause flowers to fall off. Additionally, aphids produce a sticky residue called honeydew [14], which encourages the growth of black sooty mold on the plants.	
Leaf Wilt	Leaf wilt in eggplant, often caused by verticillium wilt [15], is a fungal disease that resides in the soil. Initially, it manifests with yellowing at the edges and tips of the leaves. As the disease advances, the entire leaf turns yellow and may spread throughout the plant [16]. This fungal infection disrupts the plant's ability to transport water and nutrients [7], leading to symptoms of wilting and stunted growth. Early identification and management are crucial to mitigate the impact of verticillium wilt on eggplant crops.	
Fresh Eggplant Leaf	Fresh eggplant leaves are typically large with slightly serrated edges [6], giving them a jagged appearance. When young, they may appear lighter in color and have a softer texture. As they mature, the leaves become darker green and more robust [7]. The overall shape and size of the leaves remain consistent throughout the plant's growth.	
Defect Eggplant	Defect eggplants exhibit noticeable physical abnormalities or signs of deterioration. These include irregular or misshapen forms, surface scars, blemishes, and discoloration that deviate from the healthy appearance. Their skin may appear dull, wrinkled, or uneven in texture, and they often show soft spots, mushiness, or inconsistent firmness when handled. Such features can result from handling damage, pest attacks, or disease-related issues.	
Fresh Eggplant	Fresh eggplants are characterized by a uniform, healthy appearance with smooth, glossy, and vibrant skin. They are firm to the touch without soft spots or blemishes. Color varies by variety—from deep purple to lavender, white, striped, or green—but should be consistent and free of discoloration or dullness. Shapes typically range from oval to elongated, and their size depends on the cultivar, commonly between 6 and 12 inches in length. A natural waxy sheen on the skin is a sign of freshness [17].	

**Fig. 2:**
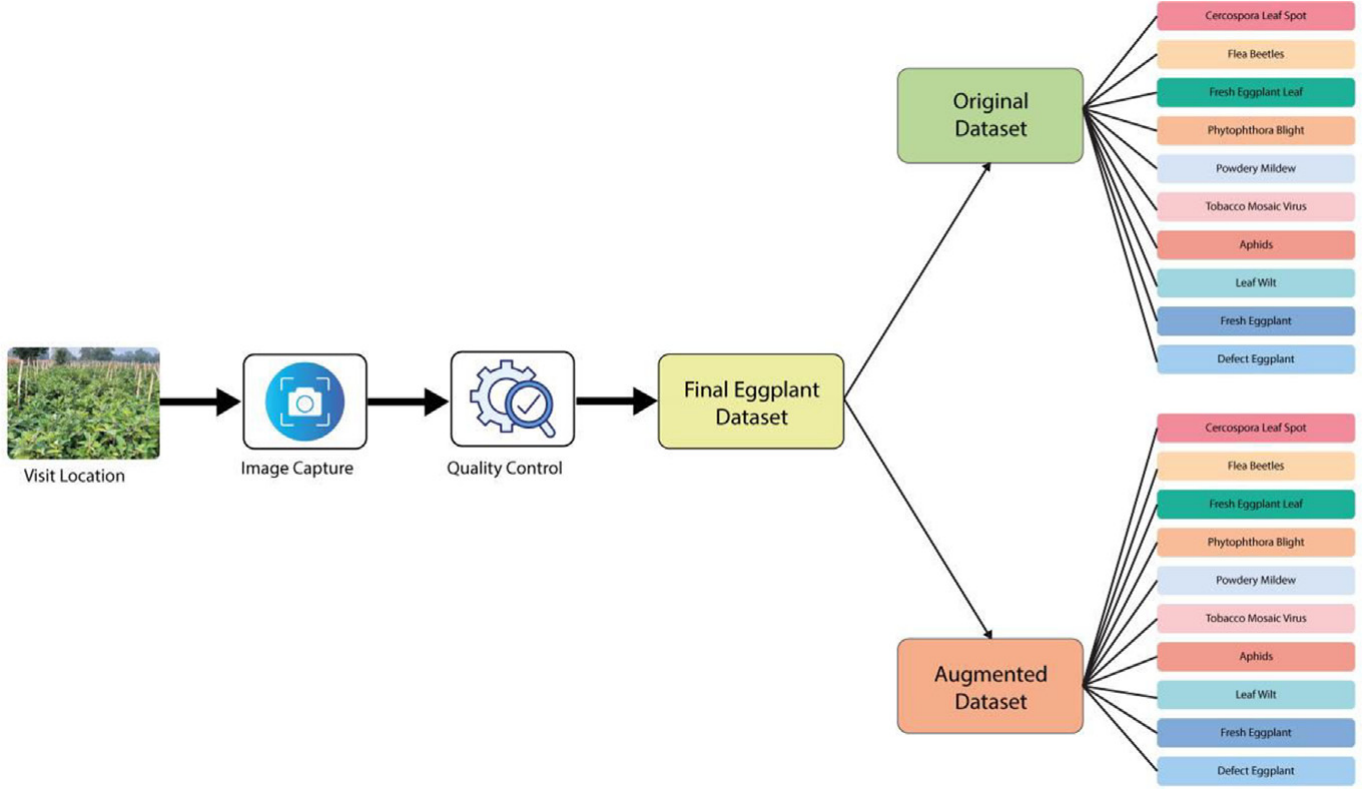
The visual representation of the organizational structure of the dataset.

This paper presents the Eggplant Dataset to support research in agricultural technology and plant pathology. Fig. 2 illustrates how the dataset was created and organized, from field visits to capture images of eggplant plants in their natural settings to rigorous quality control procedures that guarantee the reliability and accuracy of this dataset.

The diagram outlines the division of the dataset into two main subcategories:

**Original Dataset:** High-resolution images of various eggplant diseases with detailed annotations are contained therein.

**Augmented Dataset:** This is the augmented dataset resulting from applying a range of augmentation techniques-flipping, rotation, and adding noise-operated on the original dataset. This increased robustness and variability make this dataset ideal for a wide variety of machine learning tasks.

Both categories represent images of each common eggplant disease, such as Cercospora Leaf Spot, Flea Beetles, Phytophthora Blight, Powdery Mildew, Tobacco Mosaic Virus, and others. This well-balanced dataset will definitely be useful in the development of state-of-the-art machine learning models for diseases detection and plant protection.

### 3.1 Applications and benefits of the eggplant dataset

**Advancement in Plant Pathology Research:** The dataset provides a detailed collection of high-resolution images and annotations of various eggplant diseases. Researchers can analyze disease progression, symptom development, and environmental influences, leading to deeper insights into disease management strategies.

**Development of AI-Driven Disease Detection Tools:** Machine learning models trained on this dataset can enable accurate and automated detection of eggplant diseases. These tools can assist farmers in early disease diagnosis, facilitating timely intervention and minimizing crop losses.

**Enhancement of Precision Agriculture:** Integrating AI models trained on the dataset with IoT devices and drones enables real-time monitoring of crop health. This supports precision agriculture practices by optimizing resource allocation and improving overall farm productivity.

These applications highlight the dataset's multifaceted utility in advancing agricultural research, technological innovation, and practical farming solutions in eggplant cultivation.

Robust quality control measures and validation techniques implemented during the data collection phase guarantee the dataset's reliability and accuracy. By facilitating seamless integration with machine learning frameworks, the dataset aids researchers and developers in advancing plant pathology research and improving agricultural practices. Ultimately, this dataset seeks to enhance the resilience of eggplant cultivation against common diseases, contributing to sustainable agricultural development.

## 4 Experimental Design, Materials and Methods

The dataset for this study was gathered from two distinct locations in Bangladesh: Dattapara, Ashulia, Dhaka, and Horinadundu, Jhenaidah Sadar, between March and May 2024. These sites were strategically selected based on prominence in eggplant cultivation, diversified agricultural settings, and accessibility to collaborate effectively with local farmers in field observations and data collection. These areas are well-known for their high eggplant production and significantly contribute to the regional market supply. In fact, two locations had to be chosen since not all types of diseases were present in one single field; for comprehensive data collection of the targeted diseases, data needed to be collected from more than one field. Images of the leaves of the eggplants were captured using Redmi Note 11 Pro Plus and Samsung S21 phones, each having a resolution of 800 × 800 pixels, totalling 3116 high-resolution images. These devices were picked for their portability and advanced features in imaging, such as the 108 MP main camera on the Redmi Note 11 Pro Plus, with a 64 MP telephoto combined with a 12 MP wide-angle lens for the Samsung S21, so that sharp and clear images could be guaranteed in variable environmental conditions. These include HDR mode and optical image stabilization, improving image clarity by reducing motion and inconsistent lighting distortion. Domain experts meticulously annotated each image to identify and classify various eggplant leaf diseases, including Cercospora Leaf Spot, Flea Beetles, Phytophthora Blight, Powdery Mildew, Tobacco Mosaic Virus, Aphids, Leaf Wilt, Fresh Eggplant, and Defective Eggplant.

Stringent quality control measures were implemented due to adverse weather conditions, such as unusually cold weather, which posed challenges like noisy backgrounds and uneven lighting. Annotations were validated using random sampling and cross-validation techniques to maintain accuracy and consistency across the dataset.

**Fig. 3:**
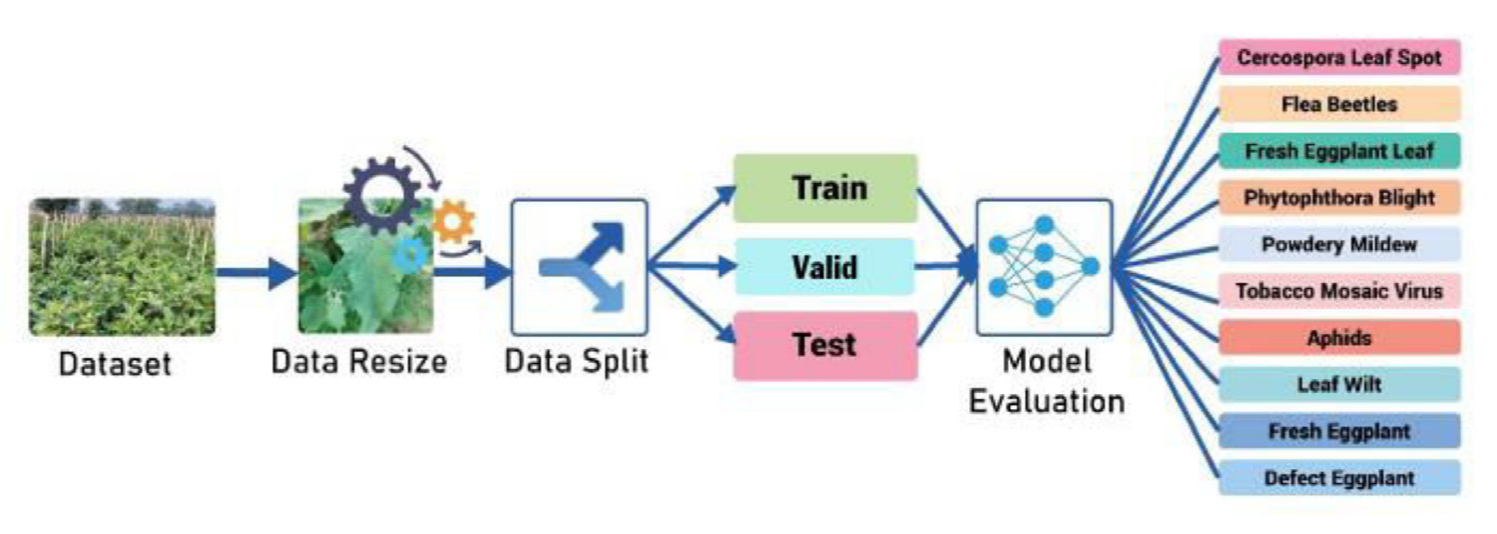
Work flow of dataset evaluation.

After completing the data collection and preprocessing steps—including resizing, normalization, and cleaning to optimize image suitability for model training—the dataset was divided into three subsets: 80 % for training, 10 % for validation, and 10 % for testing. This division follows standard machine learning practices, ensuring that the model has access to a substantial amount of data for effective learning while retaining sufficient samples for both validation and independent testing. Such a balanced split enhances the model’s generalization ability and provides reliable evaluation results. This approach is also widely adopted in agricultural image classification tasks, where the objective is to optimize both training efficiency and model assessment. The overall process of applying machine learning techniques to the eggplant dataset is illustrated in Fig. 3.

Deep learning models were employed to develop a robust framework capable of identifying and classifying various conditions affecting eggplant leaves as depicted in the dataset. These conditions include Cercospora Leaf Spot, Flea Beetles, Phytophthora Blight, Powdery Mildew, Tobacco Mosaic Virus, Aphids, Leaf Wilt, Fresh Eggplant, and Defective Eggplant. Model performance was evaluated using metrics such as accuracy, precision, and recall on the validation dataset to assess its effectiveness in disease detection and classification.

### 4.1 Camara specification

The dataset was meticulously collected using two smartphones renowned for their advanced camera capabilities: the Redmi Note 11 Pro Plus and the Samsung S21. The Redmi Note 11 Pro Plus features a primary 108 MP camera with an f/1.9 aperture, excelling in capturing fine details even in challenging lighting conditions. Its 8 MP ultra-wide lens provides an expansive 118° field of view, ideal for landscapes, while the 2 MP macro lens highlights intricate close-up details. A 2 MP depth sensor enhances portrait photography with depth and bokeh effects. Similarly, the Samsung S21 employs a sophisticated camera setup with a high-resolution main sensor and features like Dual Pixel PDAF and OIS for precise focusing and stabilization. Its 12 MP ultra-wide lens and 64 MP telephoto sensor enable capturing expansive scenes and detailed zoom shots respectively. Both smartphones contribute diverse high-resolution images essential for training and validating deep learning models in plant pathology research, ensuring accurate disease detection and classification.

### 4.2 Data augmentation

Data augmentation is a crucial technique in machine learning, particularly within deep learning and computer vision applications. It involves creating augmented versions of existing training data by applying various transformations such as rescaling, rotation, adjusting width and height, shearing, zooming, horizontal flipping, and brightness adjustments. These transformations diversify the dataset, serving multiple critical purposes.

These augmentations are essential for improving the model's ability to generalize to unseen examples and handle real-world variations, which is particularly important in agriculture. Agricultural datasets often face challenges like limited data availability, inconsistent image quality, varying environmental conditions, and diverse plant growth stages. The implementation of augmentation techniques helps overcome these challenges by simulating different real-world scenarios.We employed several methods: flipping to create mirror images, 90-degree rotation for orientation diversity, Gaussian noise to simulate varying lighting conditions, height shifting by 10 % to address positional variations, brightness shifting within a range of 0.5 to 1.5, zooming with factors between 1 and 1.5 for scale variations, and shearing with factors between 0.1 and 0.5 for perspective distortions. Implemented using OpenCV and NumPy, these techniques significantly increased the dataset's size and diversity. The specific parameters used for each augmentation technique are detailed in the following Table 2.

**Table 2:** Augmentation strategies for dataset enhancement.

Augmentation Technique	Parameter	Value/Range
Flipping	Direction	Horizontal
Rotation	Angle	90 degrees clockwise
Gaussian Noise	Mean, Standard Deviation	0, 25
Height Shifting	Shift Amount	10 % of image height
Brightness Shifting	Brightness Factor	0.5 to 1.5
Zooming	Zoom Factor	1 to 1.5
Shearing	Shear Factor	0.1 to 0.5

Data augmentation helps mitigate overfitting by exposing the model to a wider range of scenarios and variations present in real-world data. Overfitting occurs when a model becomes too specialized to the training data, leading to poor generalization on unseen data. By introducing augmented images that simulate different lighting conditions, orientations, and perspectives, data augmentation improves the model's ability to generalize and enhances its robustness.

Additionally, data augmentation effectively increases the size of the training dataset without the need for additional data collection. This is particularly advantageous when original data is limited, a common challenge in agriculture, where data acquisition can be time-consuming and costly. Augmentation providing more instances for the model to learn from and thereby improving its performance.

**Fig. 4:**
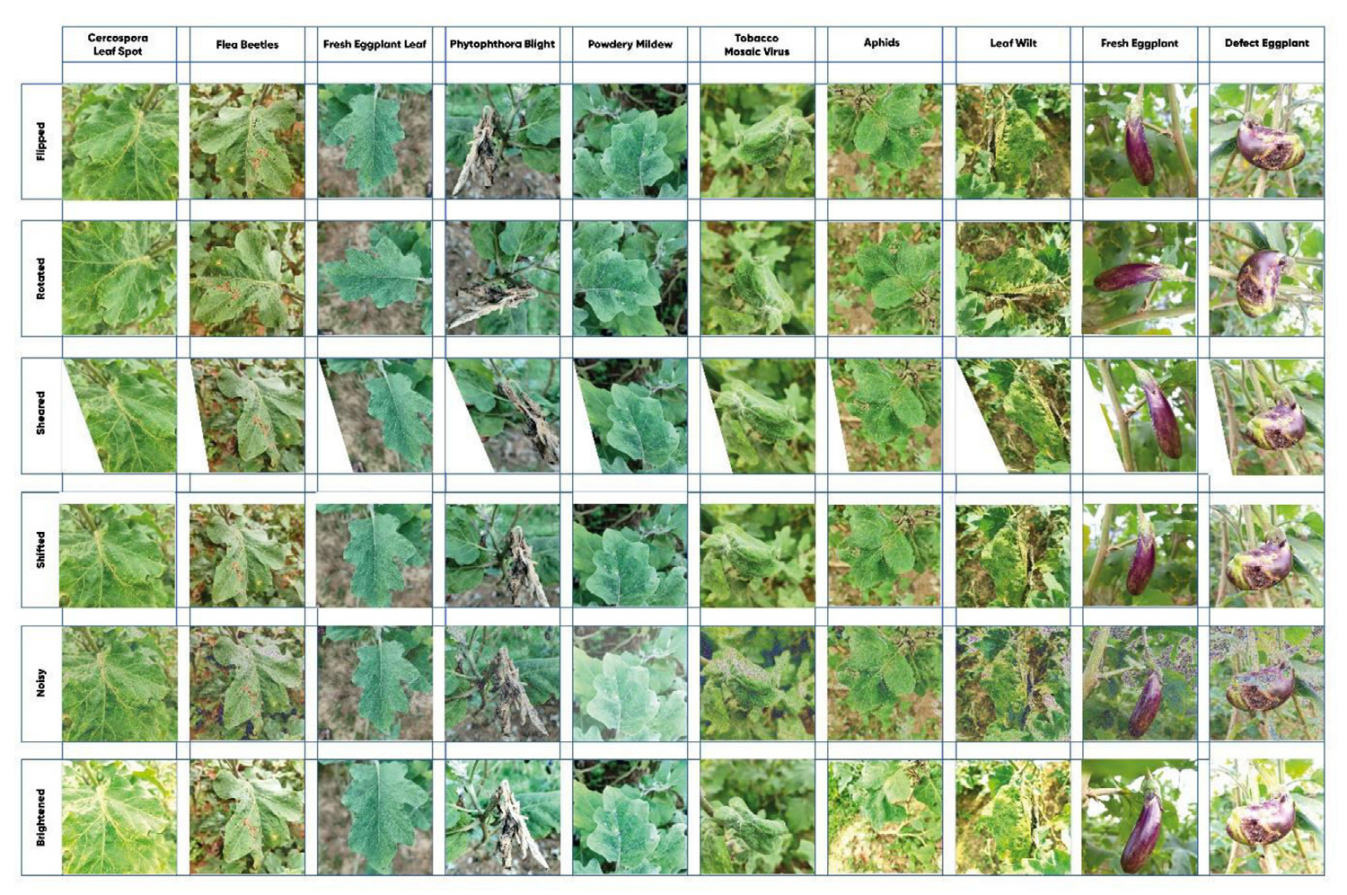
Augmented images from the eggplant dataset.

Fig. 4 illustrates the various data augmentation techniques applied to the eggplant dataset, aimed at enhancing its diversity and robustness. To achieve this, we employed augmentations including flipping, rotating, shearing, shifting, adding noise, and adjusting brightness, as depicted in each row of the figure. These techniques introduced variations to the original images, creating different orientations, perspectives, and lighting conditions. The augmented dataset features conditions such as Cercospora Leaf Spot, Flea Beetles, and Powdery Mildew. This approach significantly improves the training of machine learning models, enabling accurate detection and classification of eggplant diseases across diverse and realistic scenarios.

As shown in Table 3, the dataset initially had class imbalance, such as only 85 original images for Leaf Wilt compared to 573 for Fresh Eggplant. To address this, targeted augmentation was applied to equalize each class to 1000 samples, ensuring balance across all categories. While augmentation improves dataset balance, we also acknowledge that some classes such as Leaf Wilt and Powdery Mildew exhibit visually similar symptoms, which can influence classification accuracy.

**Table 3:** Statistics of the eggplant disease.

Class Name	Number of original images	Number of augmented images
Cercospora Leaf Spot	133	1000
Flea Beetles	353	1000
Fresh Eggplant Leaf	256	1000
Phytophthora Blight	423	1000
Powdery Mildew	213	1000
Tobacco Mosaic Virus	406	1000
Aphids	224	1000
Leaf Wilt	85	1000
Fresh Eggplant	573	1000
Defect Eggplant	450	1000
**Total**	**3116**	**10,000**

In the context of the Eggplant Dataset, as depicted in Fig. 4 and detailed in Table 3, data augmentation significantly enriched the dataset. The original 3116 images were augmented to a total of 10,000 images across various disease classes. This augmentation process involved carefully adjusting parameters such as rotation, shift, shear, zoom, and brightness, ensuring a diverse and representative dataset suitable for training deep learning models.

Ultimately, the augmented dataset not only enhanced the model's training efficacy in recognizing and classifying eggplant leaf diseases but also boosted overall model performance and reliability. Data augmentation remains a fundamental technique in machine learning, pivotal for advancing model capabilities in tasks like visual object recognition and disease classification in agricultural contexts.

### 4.3 Model validation

In this work, we propose a hybrid architecture that integrates the Convolutional Block Attention Module (CBAM) with the EfficientNetB0 backbone to evaluate the effectiveness of our model for eggplant leaf disease classification. The architecture replaces the native classification head with a custom CBAM attention block, followed by a Global Average Pooling (GAP) layer, a Dropout layer (rate = 0.25), and a Dense layer with softmax activation for final classification. The EfficientNetB0 model, initialized with pre-trained ImageNet weights, serves as the feature extractor. Importantly, the weights of EfficientNetB0 are frozen during the initial training phase to stabilize learning, while the newly added layers are trained to adapt to the specific task at hand.

**Fig. 5:**
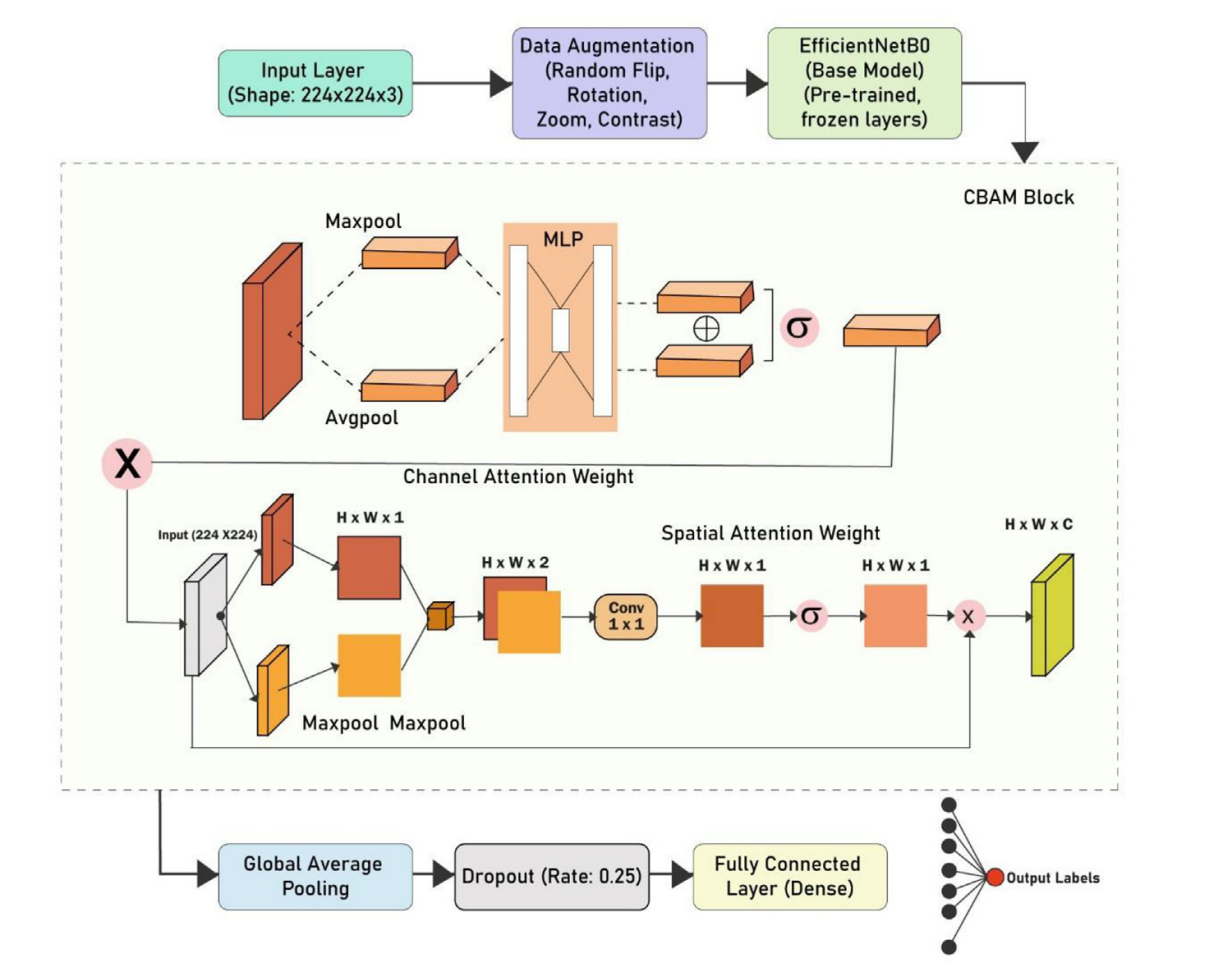
Architecture of the proposed CBAM–EfficientNetB0-based model for eggplant leaf disease classification.

The CBAM mechanism, which is strategically integrated into the architecture, fine-tunes feature maps at both the channel and spatial levels. This mechanism allows the model to focus on the most relevant features in eggplant leaf images that are indicative of disease. Specifically, the channel attention block applies a multi-layer perceptron (MLP) architecture to aggregate global information, followed by max pooling to generate the channel attention weights. The spatial attention block further refines these feature maps by applying convolutional operations to calculate the spatial attention weights, directing the network's focus on the region’s most relevant for classification (Fig. 5).

During training, we adopted a two-stage strategy. In the first stage, the EfficientNetB0 base was frozen, and only the newly added layers were trained to stabilize learning. In the second stage, the top 20 % of convolutional layers were unfrozen for fine-tuning while keeping Batch Normalization layers frozen to preserve pre-trained statistics. The Adam optimizer was employed with an initial learning rate of $1\;\times 10^{-3}$, later reduced to $1\;\times 10^{-4}$ during fine-tuning. A learning rate scheduler, early stopping, and model checkpointing were integrated to prevent overfitting and ensure stable convergence. Training was performed for up to 30 epochs with a batch size of 32. To handle class imbalance, class weights were applied based on sample distribution across categories. Additionally, data augmentation techniques such as random flipping, rotation, zoom, and contrast adjustment were applied to improve robustness against real-world variability.

Our proposed model achieved 98.70 % classification accuracy, which substantially outperformed baseline models including VGG16, VGG19, and ResNet50. Specifically, ResNet50 achieved only 32.60 %, VGG16 achieved 73.21 %, and VGG19 achieved 68.81 %. To further validate the robustness of our proposed model, we applied 5-fold cross-validation. The results are summarized as Table 4.

**Table 4:** 5-fold cross-validation results of the proposed CBAM–EfficientNetB0-based Model.

Fold	Validation Accuracy	Validation Loss
1	0.9600	0.1170
2	0.9413	0.1971
3	0.9619	0.1285
4	0.9794	0.1018
5	0.9894	0.1007

To assess the effectiveness of the model, we employed several evaluation metrics: accuracy, precision, recall, and F1-score. These metrics offer comprehensive insights into the model's classification performance from different perspectives, ensuring a robust evaluation. The softmax function is typically applied at the output layer of classification to convert raw scores into probabilities. For a given class $c_{,}$ the softmax function is defined as:

(1) $$softmax\left(z_{i}\right)=\frac{e^{z_{i}}}{\sum_{j=1}^{K}e^{zj}}$$

where $z_{i}$ is the raw score for class $i_{,}$ and K is the total number of classes. This normalization ensures that the sum of probabilities across all classes equals 1, providing a probabilistic interpretation of the model's outputs. The confusion matrix played a crucial role in our evaluation, providing a detailed breakdown of the model's predictions against ground truth labels across various disease categories. This visual representation enabled us to analyze classification accuracy and identify specific error patterns.

### 4.4 Key metrics

**Accuracy:** Measures the overall correctness of the model's predictions:

(2) $$Accuracy=\frac{\sum_{i=1}^{K}\;True\;Positive_{i}\;}{\sum_{i=1}^{K}\left(True\;Positive_{i}+True\;Negative_{i}+False\;Positive_{i}+False\;Negative_{i}\right)}$$

**Macro Precision, Recall, and F1-Score:** These metrics treat each class equally by calculating the metric independently for each class and then taking the average:

(3) $$Macro\;Precesion=\;\frac{1}{K}\sum_{i=1}^{K}\frac{True\;Positive_{i}}{\left(True\;Positive_{i}+\;False\;Positive_{i}\right)}$$

(4) $$Macro\;Recall=\;\frac{1}{K}\sum_{i=1}^{K}\frac{True\;Positive_{i}}{\left(True\;Positive_{i}+\;False\;Negative_{i}\right)}\;$$

(5) $$Macro\;F1-Score=\;\frac{1}{K}\sum_{i=1}^{K}\frac{2\;\times Precision_{i}\times Recall_{i}}{Precision_{i}+\;Recall_{i}}$$

**Micro Precision, Recall, and F1-Score:** These metrics aggregate the contributions of all classes to compute the average:

(6) $$Micro\;Precesion=\;\frac{\sum_{i=1}^{K}True\;Positive_{i}}{\sum_{i=1}^{K}\left(True\;Positive_{i}+\;False\;Positive_{i}\right)}$$

(7) $$Micro\;Recall=\;\frac{\sum_{i=1}^{K}True\;Positive_{i}}{\sum_{i=1}^{K}\left(True\;Positive_{i}+\;False\;Negative_{i}\right)}$$

(8) $$Micro\;F1-Score=\frac{2\;\times Micro\;Precision\times Micro\;Recall\;}{Micro\;Precision+\;Micro\;Recall}$$

**Weighted Precision, Recall, and F1-Score:** These metrics account for the support (number of true instances) of each class:

(9) $$Weighted\;Precesion=\;\frac{\sum_{i=1}^{K}Support_{i}\times Precision_{i}}{\sum_{i=1}^{K}Support_{i}}$$

(10) $$Weighted\;Recall=\;\frac{\sum_{i=1}^{K}Support_{i}\times Recall_{i}}{\sum_{i=1}^{K}Support_{i}}$$

(11) $$Weighted\;F1-Score=\;\frac{\sum_{i=1}^{K}Support_{i}\times F1-Score_{i}}{\sum_{i=1}^{K}Support_{i}}\;$$

**Fig. 6:**
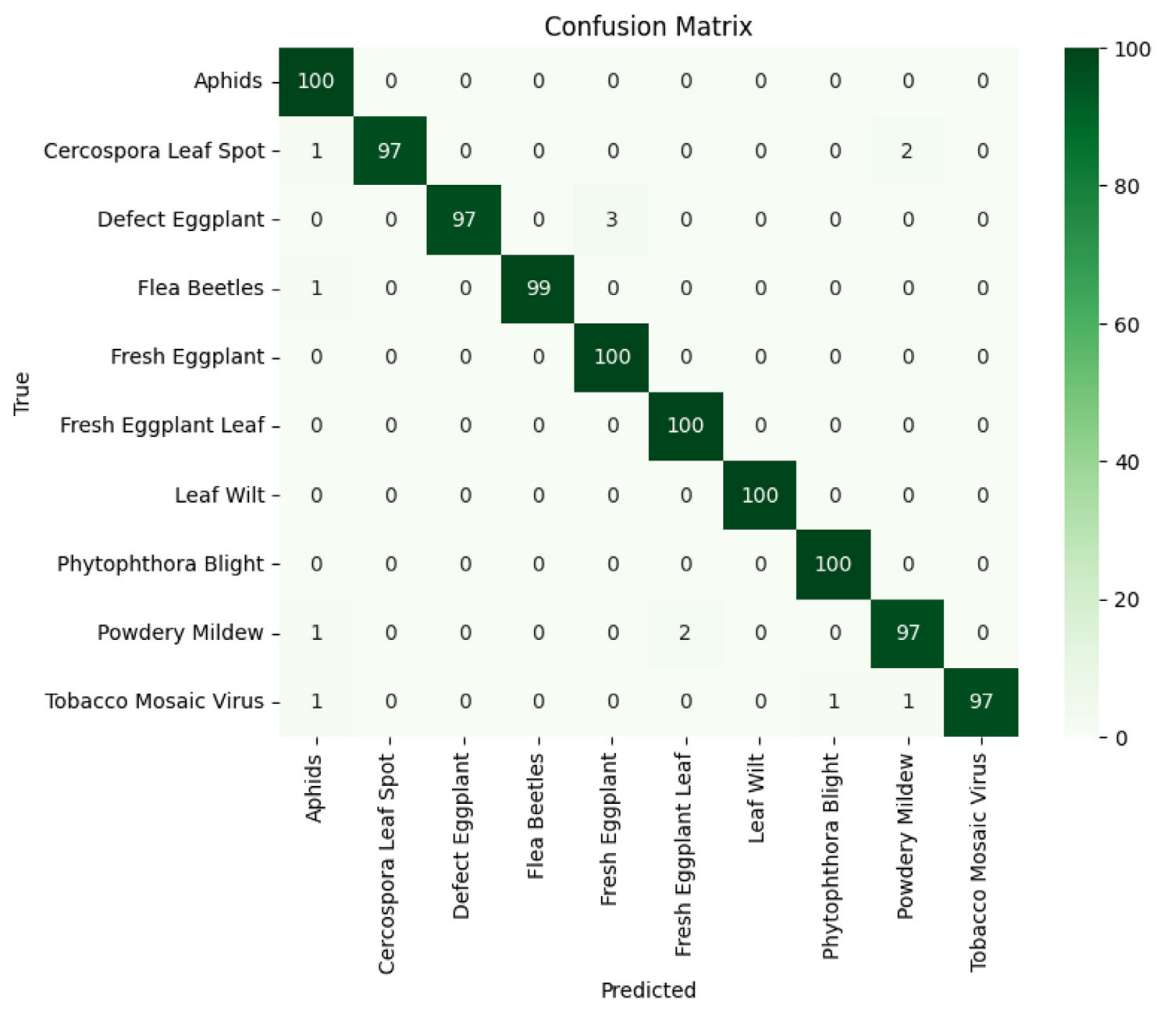
Confusion matrix of proposed CBAM–EfficientNetB0-based model on eggplant disease classification.

Fig. 6 present the confusion matrices for the proposed CBAM–EfficientNetB0-based model, respectively, highlighting their classification performance across ten eggplant disease classes.

**Fig. 7:**
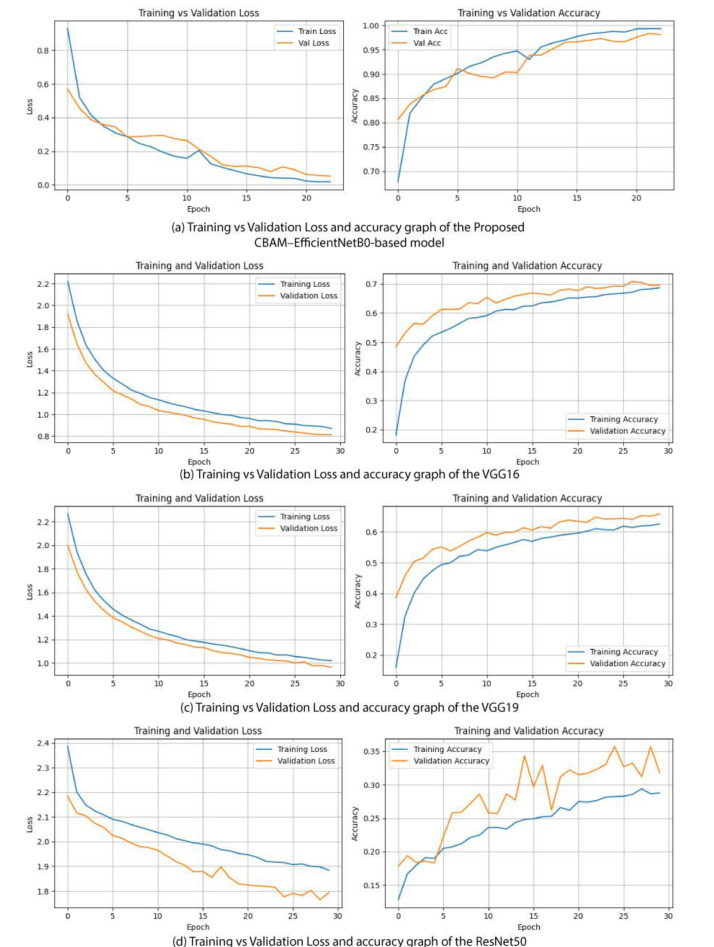
Visualization of training vs validation accuracy and loss graph.

Fig. 7(a–d) present the training and validation accuracy and loss curves for the proposed CBAM–EfficientNetB0 model and baseline models (VGG16, VGG19, and ResNet50) over 30 epochs. The proposed CBAM–EfficientNetB0 model (Fig. 7(a)) shows smooth convergence with closely aligned training and validation curves, indicating strong generalization and minimal overfitting. VGG16 (Fig. 7(b)) achieves reasonable accuracy but exhibits early plateauing and moderate overfitting, while VGG19 (Fig. 7(c)) displays greater fluctuations and instability in validation performance. In contrast, ResNet50 (Fig. 7(d)) demonstrates poor convergence, with validation accuracy remaining low and inconsistent. Overall, the proposed CBAM–EfficientNetB0 significantly outperforms the baseline models in terms of stability, generalization, and classification performance.

Following our evaluation in Table 5, the CBAM EfficientNetB0-based model achieved an outstanding accuracy of 98.70 %, demonstrating its exceptional ability to distinguish between various eggplant diseases. This high accuracy highlights the model's potential for practical deployment in agriculture, with precision, recall, and F1-score metrics further validating its reliability for precision agriculture applications, particularly for early disease detection and management. In comparison, the ResNet50 model, while still performing reasonably well with an accuracy of 32.60 %, showed significantly lower precision and recall values across most classes, which can be attributed to its architectural limitations. Additionally, the VGG16 and VGG19 models, while showing better performance than ResNet50 with accuracy rates of 73.21 % and 68.81 %, respectively, still fell short of the CBAM EfficientNetB0 model in terms of overall classification ability. While VGG16 and VGG19 performed reasonably well for certain classes like Fresh Eggplant Leaf, their architectures lack the advanced features, such as the attention mechanism in CBAM EfficientNetB0, which contribute to its superior performance in complex disease classification tasks.

**Table 5:** Comparison of model evaluation metrics (classification report and accuracy for DenseNet201 and ResNet50).

Model	Class Name	Precision	Recall	F1-score	Accuracy
	Cercospora Leaf Spot	1.00	0.970	0.984	98.70 %
	Flea Beetles	1.00	0.99	0.995	
	Fresh Eggplant Leaf	0.9804	1.00	0.9901	
	Phytophthora Blight	0.9901	1.00	0.9950	
Proposed CBAM EfficientNetB0 based model	Powdery Mildew	0.970	0.970	0.970	
	Tobacco Mosaic Virus	1.00	0.97	0.9848	
	Aphids	0.9615	1.00	0.9804	
	Leaf Wilt	1.00	1.00	1.00	
	Fresh Eggplant	0.9709	1.00	0.9852	
	Defect Eggplant	1.00	0.970	0.9848	
	Cercospora Leaf Spot	0.9569	0.886	0.8462	
	Flea Beetles	0.8345	0.47	0.35	
	Fresh Eggplant Leaf	0.68	0.39	0.68	
	Phytophthora Blight	0.80	0.68	0.74	
VGG16	Powdery Mildew	0.84	0.72	0.77	73.21 %
	Tobacco Mosaic Virus	0.84	0.72	0.77	
	Aphids	0.80	0.68	0.74	
	Leaf Wilt	0.60	0.74	0.66	
	Fresh Eggplant	0.84	0.72	0.77	
	Defect Eggplant	0.59	0.56	0.67	
	Cercospora Leaf Spot	0.55	0.64	0.73	
	Flea Beetles	0.73	0.77	0.63	
	Fresh Eggplant Leaf	0.82	0.77	0.79	
	Phytophthora Blight	0.57	0.62	0.76	
VGG19	Powdery Mildew	0.70	0.66	0.68	68.81 %.
	Tobacco Mosaic Virus	0.81	0.65	0.59	
	Aphids	0.71	0.50	0.69	
	Leaf Wilt	0.70	0.63	0.74	
	Fresh Eggplant	0.79	0.78	0.63	
	Defect Eggplant	0.80	0.51	0.70	
	Cercospora Leaf Spot	0.25	0.38	0.42	
	Flea Beetles	0.48	0.45	0.36	
	Fresh Eggplant Leaf	0.34	0.47	0.24	
	Phytophthora Blight	0.44	0.25	0.42	
ResNet50	Powdery Mildew	0.23	0.21	0.45	32.60 %
	Tobacco Mosaic Virus	0.28	0.31	0.20	
	Aphids	0.42	0.31	0.48	
	Leaf Wilt	0.22	0.23	0.25	
	Fresh Eggplant	0.20	0.34	0.40	
	Defect Eggplant	0.23	0.27	0.43	

## Limitations

Our study is the lack of samples collected from countries other than Bangladesh, which may restrict the dataset's representativeness and generalizability to a broader geographical context. Moreover, there might be a seasonal and environmental bias because of the particular period in which data was collected. Data collection in all seasons and varied environmental conditions can eliminate this bias. It occurs when data is collected from only one season, causing the model to overfit to specific environmental conditions and perform poorly in other seasons. To address these limitations, future work should focus on multi-seasonal data collection throughout the year and extend coverage to different geographical regions, both within and outside Bangladesh. Incorporating images from diverse climates and agricultural practices would improve the robustness of the model. Furthermore, regularization techniques (e.g., dropout, early stopping), ensemble methods, and cross-validation should be applied to mitigate overfitting to season-specific features. Longitudinal studies and transfer learning approaches could also enhance the adaptability of models to other crops within the Solanaceae family, such as tomatoes and peppers, supporting broader precision agriculture applications.

## Ethics Statement

The research conducted for this paper did not involve any experiments or studies with humans or animals as subjects. The dataset used consists solely of images obtained by the authors, adhering to ethical guidelines. All consulted datasets are publicly accessible, and proper citation guidelines have been followed.

## Credit Author Statement

**Md. Asraful Sharker Nirob:** Conceptualization, Methodology, Writing – original draft, Data curation. **Prayma Bishshash:** Conceptualization, Visualization, Data curation, Writing. **Mariyam Bin Ayan:** Validation, Data curation, Writing. **Tania Khatun:** Supervision, Formal analysis, Writing – review & editing. **Shayla Sharmin:** Supervision, Formal analysis. **Md Zahid Hasan:** Supervision, Formal analysis, Writing – review & editing. **Mohammad Shorif Uddin:** Supervision – Data Collection.

## Data Availability

Mendeley DataEggplant Dataset: A Comprehensive Dataset for Agricultural Research and Disease Detection (Original data).
